# Trauma Trends During the Initial Peak of the COVID-19 Pandemic in the Midst of Lockdown: Experiences From a Rural Trauma Center

**DOI:** 10.7759/cureus.9811

**Published:** 2020-08-17

**Authors:** Heather X Rhodes, Kirklen Petersen, Saptarshi Biswas

**Affiliations:** 1 Trauma, Grand Strand Medical Center, Myrtle Beach, USA

**Keywords:** trauma, corona virus, covid-19

## Abstract

Background

As the early peak phase in the coronavirus outbreak has intensified, stay at home mandates were advised requiring individuals to remain home to prevent community transmission of the disease. Further mandates escalated isolated environments such as school closures, social distancing, travel restrictions, closure of public gathering spaces, and business closures. As citizens were forced to stay home during the pandemic, the crisis created unique trends in trauma referrals, which consisted of atypical trends in injuries related to trauma.

Methods

A retrospective review of all trauma registry patients presenting to a rural American College of Surgeons (ACS) verified Level I trauma center with associated trauma activation before and during the Coronavirus 2019 (COVID-19) pandemic, integral dates January 1, 2020, to May 1, 2020. A comparison was made regarding trauma trends based on the previous year (January 1, 2019, to May 1, 2019). The data collected included patient characteristics, grouping by trauma activation, injury type, injury severity score (ISS), alcohol screen, drug screen, and mode of injury.

Results

A statistically significant increase was found largely among males (p = 0.02) with positive alcohol screens (p < 0.001). The statistically significant mode of injury among this trauma population included falling, jumping, pushed (p = 0.02); self-harm-jump (p = 0.01); assault (p = 0.03); and assault with sharp object (p = 0.036).

Conclusions

Although overall trauma volume was reduced preceding and during the COVID-19 stay at home mandates, a significant increase in specific trauma trends were observed, such as falls, jumps, and pushed; self-harm-jumps; assaults; and assaults with sharp objects. Largely, the trauma trends were among men with higher levels of alcohol than previously reported.

## Introduction

As a result of the COVID-19 pandemic and subsequent stay at home mandates, trauma trends have drastically changed. Many people have altered their daily routines in response to the novel coronavirus outbreaks, and these changes have brought about atypical trends in injuries related to trauma. There has been a decline in the number of acute trauma referrals, admissions, operations, and aerosolizing anesthetic procedures since implementing the stay at home mandates [1]. At a Level II trauma center in New Hampshire, significant decreases in overall trauma admissions have been as high as 57.4% [2]. Counties have noted up to 4.8-fold decrease in trauma activations since the shelter-in-place orders went into effect [3]. The restricted travel mandates have had significant impacts on motor vehicle collisions (MVC). Trauma centers located in Florida, New York, and Massachusetts have reported a significant downward trend in MVCs [4]. After reviewing trauma admissions from 2017 to 2020, February to April, a New Hampshire Level II trauma center reported an 80.5% reduction in MVCs [2]. Pedestrian vehicle accidents have decreased as well [5].

There has also been a 43% reduction in all injury-related admissions [6]. Previous reports include significant reductions in major injury (50% reduction), males (50% reduction), and children aged 0-14 years (48% reduction) [6]. In a regional emergency department (ED) in South Africa, categories showing major decreases include assault and gunshot wounds [5]. Among two tertiary orthopedic trauma centers in Iran, most injuries were seen in 21- to 40-year-old patients [7]. The majority of injuries during the lockdown at a Level I trauma center in New Zealand occurred at home, predominantly falls [6]. The most common types of injury included low energy and blunt trauma [3,7]. Since the lockdown, differences have developed in the classes and rates of trauma experienced by men versus women. Women have been more affected by low energy trauma, whereas men have been more affected by sharp injuries and blunt trauma during work [7]. This observation could be associated with better implementation of stay-at-home mandates and a reduction of risk-taking by women when compared to men [7]. Overall, the quarantine has been safer for women, as there has been an increase in the men to women ratio of risk-taking traumatic injuries [7]. A reduction in sports-related ligamentous injuries has also been noted, with rates as low as 2% [7].

With shelter-in-place orders in effect and fewer people out in their communities, one might expect fewer opportunities for injury [2]. The reduction in injuries may reduce the strain on the healthcare system by decreasing hospital admissions from trauma and reducing virus transmission [3]. Despite overall reductions in the number of adult and pediatric trauma cases during the COVID-19 pandemic, specific injury mechanisms have become more common. For example, injuries associated with using tools at home have significantly increased [5,8]. Since the beginning of the stay at home mandates, few studies have investigated specific types of trauma, those most likely to suffer from traumatic injury, and factors related to trauma such as substance abuse. This study aims to present recent trends in trauma that have developed during the COVID-19 pandemic.

## Materials and methods

A retrospective study was conducted on prospectively collected trauma registry data on injured patients of all age groups, and injury severities admitted sequentially to an American College of Surgeons (ACS) verified Level I trauma center in South Carolina before and after community lockdown in response to the COVID-19 pandemic. The two groups consisted of trauma admissions, which were reported during corresponding timeframes within 2019 and 2020. Four months of trauma activation data were collected preceding lockdown and early peak phase of COVID-19 with groupings according to the mode of injury, alcohol and drug screen, and mortality codes, inclusive months January 1, 2020, to May 1, 2020. A comparison was made between trauma patients during the identified study period and the previous year (January 1, 2019, to May 1, 2019). Two weeks before the lockdown (March 24, 2020, to April 7, 2020) were further evaluated for potential community behavior within the pre-lockdown phase when national alert levels were escalating. Further, an investigation of trauma activations during the COVID-19 lockdown was conducted during the inclusive months of April 8, 2020, to May 1, 2020. An IRB exempt determination facilitated the data extraction from an integrated organizational repository and analysis. Continuous variables were compared using the Wilcoxon signed-rank test, whereas categorical variables were compared using Pearson’s chi-square test of proportion, as appropriate in SPSS software (SPSS, Inc., Chicago, USA). Demographic characteristics included age, race, and gender. The patients were grouped by trauma activation, injury type, injury severity score, alcohol screen, drug screen, and mode of injury. The mode of injury group was stratified into 24 specific sub-groupings by mechanism, such as assault. Injuries, causes, and procedures were coded using ICD10; additional diagnostic and injury severity scoring (ISS) was done using the automatic identification system (AIS). 

## Results

A total volume of 783 trauma-activated patients presented to the emergency department preceding lockdown, and during the early peak phase of COVID-19, inclusive months January 1, 2020, to May 1, 2020 (Table 1). Respectively, the previous year (January 1, 2019, to May 1, 2019) captured a total of 1011 trauma admissions. A closer look at trauma volume trends two weeks preceding the lockdown (March 24, 2020, to April 7, 2020) identified 105 admissions. In comparison, the preceding year (March 24, 2019, to April 7, 2019) captured a total of 150 trauma patient admissions. During the early peak phase of the outbreak and lockdown (April 8, 2020, to May 1, 2020), 89 trauma patients were identified. Compared to the previous year (April 8, 2019, to May 1, 2019), 204 trauma patients were admitted to the emergency department. The overall volume of trauma patients was reduced preceding the lockdown and during the early peak phase of COVID-19 based on these above described findings.

**Table 1:** Patient Counts

	2019	2020
Total admissions	1011	783
Two weeks prior	150	105
Outbreak and lockdown	204	89

Two statistically significant patient characteristics were observed when comparing total trauma patients preceding lockdown and early peak phase of COVID-19 to the previous year, which included impacted males (p = 0.02) and positive alcohol screens (p < 0.001; Table 2).

**Table 2:** Patient Characteristics IQR: interquartile range, ISS: injury severity score, THC: tetrahydrocannabinol, TCA: tricyclic antidepressant, OPI: opioid, COC: cocaine, BZO: benzodiazepines, AMP: amphetamine.

	2019 (N = 1011)	2020 (N = 783)	P-value
Age; median [IQR] mean ± SD	64.0 [39.0, 78.0]	62.0 [36.0, 77.0]	P = 0.147
	58.6±24.1	56.9±24.4	
Race: White	82.59% (835)	83.40% (653)	P = 0.301
Black	11.47% (116)	12.26% (96)	
Other	5.93% (60)	4.34% (34)	
Gender: Male	54.8% (554)	60.3% (472)	P = 0.02
Activation type: Consult	9.20% (93)	9.32% (73)	P = 0.295
Full	24.13% (244)	27.71% (217)	
No trauma activation	16.42% (166)	14.30% (112)	
Partial	50.25% (508)	48.66% (381)	
Injury type: Blunt	91.30% (923)	89.27% (699)	P = 0.21
Penetrating	6.03% (61)	8.17% (64)	
Other and unspec	2.67% (27)	2.55% (20)	
ISS; median [IQR] mean ± SD	5.00 [2.00, 10.00]	5.00 [2.00, 10.00]	P = 0.271
	7.48±7.11	7.67±7.20	
Alcohol screen: no (confirmed by the test)	51.8% (524)	67.4% (423)	P < 0.001
No (not tested)	26.5% (268)	32.6% (205)	
Not applicable	0.4% (4)	0.0% (0)	
Yes (beyond the legal limit)	12.6% (127)	0.0% (0)	
Yes (trace levels)	8.7% (88)	0.0% (0)	
Drug screen: No (confirmed by the test)	17.11% (173)		
No (not tested)	52.32% (529)		
Not applicable	0.20% (2)		
Unknown	0.20% (2)		
Yes (illegal use drug)	8.61% (87)		
Yes (prescription drug)	21.56% (218)		
Drug type: AMP	0.89% (9)	0.51% (4)	P = 0.772
BZO	0.99% (10)	0.64% (5)	
COC	3.07% (31)	3.45% (27)	
None	31.36% (317)	30.91% (242)	
Not tested	54.40% (550)	55.81% (437)	
OPI	0.49% (5)	0.89% (7)	
TCA	0.10% (1)	0.00% (0)	
THC	8.61% (87)	7.79% (61)	
Unknown	0.10% (1)	0.00% (0)	

**Figure 1:**
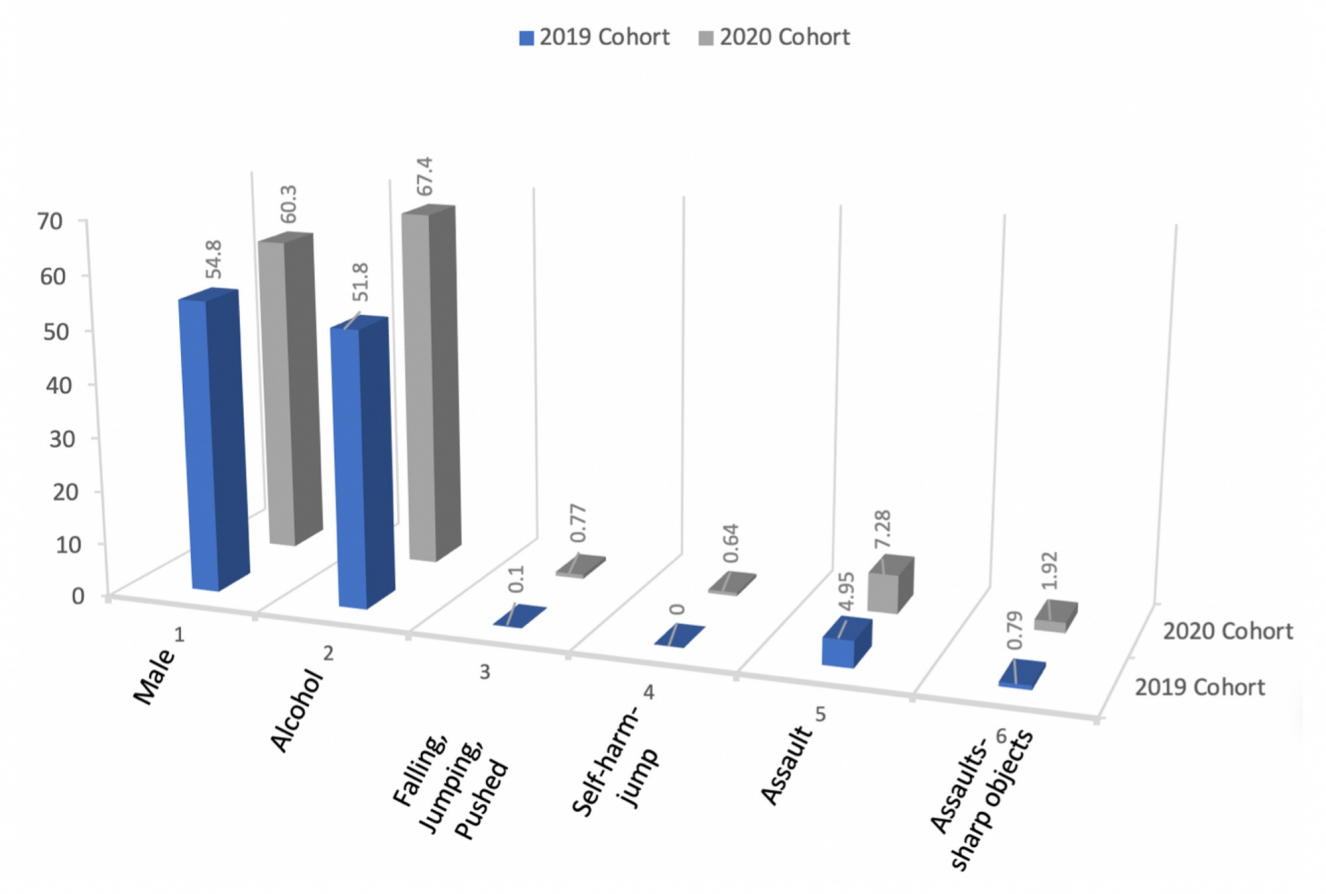
COVID-19 Trauma Trends

The comparison of mode of injury during the same comparative years revealed four statistically significant conclusions, such as falling, jumping, pushed (p = 0.02); self-harm-jump (p = 0.01); assault (p = 0.03); and assault with a sharp object (p = 0.036; Table 3; Figure 1). 

**Table 3:** Mode of Injury MVC: motor vehicle collision.

	2019 (N = 1011)	2020 (N = 783)	P-value
Gunshot wound	3.46% (35)	3.19% (25)	P = 0.753
Knife	2.27% (23)	2.30% (18)	P = 0.973
MVC	17.3% (175)	17.0% (133)	P = 0.857
Motorcycle	5.44% (55)	4.85% (38)	P = 0.578
Falling, jumping, pushed	0.10% (1)	0.77% (6)	P = 0.025
Moving object	0% (0)	0% (0)	
Suicidal ideation	0% (0)	0% (0)	
Suicide attempt	0.59% (6)	1.40% (11)	P = 0.079
Self-harm-gun	0.40% (4)	0.51% (4)	P = 0.716
Self harm-knife	0.20% (2)	0.13% (1)	P = 0.719
Self-harm-jump	0.00% (0)	0.64% (5)	P = 0.011
Undetermined intent	1.78% (18)	0.77% (6)	P = 0.064
Assault	4.95% (50)	7.28% (57)	P = 0.038
Assault-gun	1.38% (14)	2.17% (17)	P = 0.205
Assault-sharp	0.79% (8)	1.92% (15)	P = 0.036
Assault-blunt	0.99% (10)	0.77% (6)	P = 0.619
Assault-force	1.29% (13)	1.92% (15)	P = 0.286
Assault-other	0% (0)	0% (0)	
Sex abuse	0% (0)	0% (0)	
Abuse (suspected)	0.00% (0)	0.13% (1)	P = 0.256
Abuse (confirmed)	0% (0)	0% (0)	
Violence-partner	0% (0)	0% (0)	
Violence-family	0% (0)	0% (0)	
Violence-non-family	0% (0)	0% (0)	

## Discussion

The lockdown has been associated with an increased prevalence of alcohol consumption [9]. During the pandemic, among Level I trauma centers in Santa Clara County, California, 19% of patients tested positive for alcohol, and 11% tested positive for drugs [3]. This critical finding in excessive alcohol consumption may indicate an increase in the risk of contracting COVID-19 via adverse immune-related health effects and reduced diligence in social distancing measures [9]. Alcohol consumption may exacerbate multiple mechanisms of trauma and further overwhelm the healthcare system during national emergencies, such as the COVID-19 pandemic [10]. There is a need for warnings relaying excessive drinking risks during isolation to be included in public health messaging related to the pandemic [9].

Overall, this study highlights recent trends in traumatic injuries that have evolved during the early peak phase of the COVID-19 pandemic, which may indicate a diminished trauma volume; men significantly impacted; increased positive alcohol screens; increases in people that jump, fall, or are pushed; increased self-harm jumps; and increased assaults particularly with sharp objects. With the lockdowns in place and fewer opportunities for injury, many of these findings are unexpected. As a result of the novel coronavirus outbreaks globally, there has been an increased strain on the healthcare system, and many facilities have exceeded capacity and exhausted resources [9]. It is critical to recognize the mechanisms of injury that have become more prevalent during the pandemic to address and reduce future occurrences.

Limitations

As a consequence of patient chart closures' progression, an extremely contracted timeframe was available for data collection and processing. As a result, the study only captured four months of data during the early peak phase of the COVID-19 pandemic. It is envisioned that follow-up studies will use expanded timeframes and more significant patient numbers. Although the sample size of this study is limited, it is the first report on the effect of the COVID-19 lockdown on trauma trends in a rural community.

## Conclusions

Based on the study results, the timeframe preceding and during the early peak phase of COVID-19 demonstrated several significant trauma trends. Men were primarily impacted, along with positive alcohol screens. Further, the mode of trauma injuries was significantly higher among falls, jumps, and pushed; self-harm-jumps; assaults; and assaults with sharp objects. Overall trauma volume during the study period was reduced, which can be expected with the lockdown in place as fewer people are exposed to commonly observed modes of injury. We anticipate this study can determine the best methods to reduce trauma during lockdowns and ensure patients have access to the help and support they need during times, such as the COVID-19 pandemic.
